# Extensively drug-resistant (XDR) *Neisseria gonorrhoeae* cured with ceftriaxone 2 g intravenous monotherapy in Croatia, March 2026

**DOI:** 10.2807/1560-7917.ES.2026.31.21.2600422

**Published:** 2026-05-28

**Authors:** Tajana Juzbašić, Tatjana Nemeth Blažić, Nilia Volarević, Ivana Bešlić, Daniela Glažar Ivče, Ivana Ferenčak, Dragan Jurić, Ljiljana Žmak, Dolores Peruč, Susanne Jacobsson, Daniel Golparian, Ivana Božičević, Iskra Alexandra Nola, Magnus Unemo

**Affiliations:** 1 Croatian Institute of Public Health, Zagreb, Croatia; 2 Teaching Institute of Public Health of the Primorje-Gorski Kotar County, Rijeka, Croatia; 3 Department of Microbiology, School of Medicine, University of Zagreb, Zagreb, Croatia; 4 WHO Collaborating Centre for Gonorrhoea and Other STIs, National Reference Laboratory for STIs, Faculty of Medicine and Health, Örebro University, Örebro, Sweden; 5 WHO Collaborating Centre for HIV Strategic Information, Andrija Štampar School of Public Health, School of Medicine, University of Zagreb, Zagreb, Croatia; 6 Andrija Štampar School of Public Health, School of Medicine, University of Zagreb, Zagreb, Croatia; 7 Institute for Global Health, University College London, London, United Kingdom

**Keywords:** Neisseria gonorrhoeae, gonorrhoea, ceftriaxone resistance, high-level azithromycin resistance, extensively drug-resistant (XDR), Croatia

## Abstract

In early 2026 in Croatia, a male heterosexual patient of Croatian origin was diagnosed with extensively drug-resistant (XDR) gonorrhoea after unprotected vaginal intercourse with a Croatian female. The *Neisseria gonorrhoeae* HR027 isolate (genomic lineage B, MLST sequence type (ST)9903) exhibited high-level azithromycin, ceftriaxone, cefixime, benzylpenicillin, ciprofloxacin and tetracycline resistance. It had the mosaic *penA*-60.001 allele, and A2045G mutations in 23S rRNA. A single dose of ceftriaxone 2 g administered intravenously cured the infection. HR027 showed zoliflodacin and gepotidacin wild-type minimum inhibitory concentrations.

Antimicrobial resistance (AMR) in *Neisseria gonorrhoeae* (NG) compromises the effectiveness of gonorrhoea treatment in Europe and globally, with ceftriaxone high-dose monotherapy or combined with azithromycin 1–2 g being the only remaining empirical treatment options [1]. Since 2018, extensively drug-resistant (XDR)-NG isolates with high-level resistance to azithromycin plus resistance to ceftriaxone have been detected occasionally in some European countries [2-7]; the prevalence of these XDR-NG strains is, however, high in some Asian countries, especially Cambodia [8]. We report here the case of a male patient with gonococcal urethritis in Croatia, where an XDR-NG strain having ceftriaxone and high-level azithromycin resistance was isolated. The infection was cured with intravenous administration of 2 g ceftriaxone.

## Clinical gonorrhoea case description

In early 2026, a Croatian man in his late 20s presented to a general practitioner (GP) in Croatia (day 1) with dysuria. He received an empirical treatment with amoxicillin−clavulanic acid 1 g twice a day for 7 days. At a subsequent visit of the patient on day 14, when the patient remained symptomatic including a purulent urethral discharge, a urethral swab was taken yielding NG on culture (isolate HR027 (RI422)) and a NG-positive nucleic acid amplification test (NAAT; Urogenital and Resistance 12-Well; AusDiagnostics Pty Ltd, Mascot, Australia). Accordingly, gonorrhoea was diagnosed and the case notified in accordance with the communicable disease surveillance in Croatia [9,10]. The patient was heterosexual and reported unprotected vaginal intercourse with an unknown female of Croatian origin 3 days before his first visit to the GP, but no other exposure risks. The female partner was not possible to trace. The NAAT detected a ceftriaxone-resistance marker (*penA-*60.001), while it was negative for other sexually transmitted pathogens such as *Chlamydia trachomatis, Mycoplasma genitalium*, and *Trichomonas vaginalis*. No extra-genital samples (oropharyngeal or anorectal) were taken before treatment.

Treatment with a single intravenous dose of ceftriaxone 2 g was performed (day 22) [1,6]. At follow-up 14 days after ceftriaxone treatment (day 36), the patient was asymptomatic and no viable NG was detected using culture of urethral and pharyngeal swabs. A urethral swab remained low NG-positive in NAAT, which might have represented residual NG DNA from non-viable bacteria. At a second follow-up visit (day 64), the patient remained asymptomatic and a test-of-cure examining a urethral swab was negative by NAAT. Serological testing for syphilis (days 14 and 64), HIV and hepatitis C virus (day 64) was negative, but positive for hepatitis A and hepatitis B viruses due to the patient being vaccinated.

## Microbiological investigation

At the National Reference Laboratory for Gonorrhoea, Zagreb, HR027 was cultured on PolyViteX agar (bioMérieux SA, Marcy l’Etoile, France) and incubated in a humid 5% CO_2_-enriched atmosphere at 36 °C for 24 hours. Presence of β-lactamase was determined using nitrocefin test (MAST Group/Mast Diagnostica GmbH, Reinfeld, Germany). Minimum inhibitory concentrations (MICs) of eight antimicrobials were determined by Etest (bioMérieux), in accordance with the manufacturer’s instructions and interpreted according to European Committee on Antimicrobial Susceptibility Testing (EUCAST; V16.0) breakpoints, where available [11]. The MICs of the novel antimicrobials zoliflodacin [12,13] and gepotidacin [14] were determined using the agar dilution technique, in accordance with Clinical and Laboratory Standards Institute (CLSI) guidelines (M07-A9 and M100-S24, www.clsi.org) (Table) [15,16].

**TABLE:** Minimum inhibitory concentrations of 10 antimicrobials and molecular resistance determinants of a *Neisseria gonorrhoeae* XDR strain, Croatia, March 2026 (n = 1 strain)

Antibiotic	MIC in mg/L^a^	Interpretation (EUCAST V16.0 [11])	Molecular resistance determinants
Ceftriaxone	0.25	Resistant	*penA*-60.001 (mosaic encoding A311V, V316T, T483S in PBP2)
Cefixime	1	Resistant	
Azithromycin	> 256	High-level resistant	23S rRNA A2045G
Ciprofloxacin	8	Resistant	GyrA S91F and D95A, ParC S87N and E91K
Tetracycline	16	High-level resistant	*tetM*, RpsJ V57M
Benzylpenicillin	> 32	High-level resistant	*bla*_TEM-1_, *penA*-60.001
Spectinomycin	16	Susceptible	ND
Gentamicin	4	NA (wild-type MIC)	ND
Zoliflodacin	0.064	NA (wild-type MIC)	ND
Gepotidacin	1	NA (wild-type MIC)	ND

EUCAST: European Committee on Antimicrobial Susceptibility Testing; MIC: minimum inhibitory concentration; NA: not applicable due to lack of breakpoints for interpretation; ND: not detected; PBP2: penicillin-binding protein 2; STI: sexually transmitted infection; WHO: World Health Organization; XDR: extensively drug-resistant.

^a^ MIC testing was performed at the National Reference Laboratory for Gonorrhoea in Zagreb, Croatia, and results confirmed at the WHO Collaborating Centre for Gonorrhoea and Other STIs, Sweden, where also zoliflodacin [12,13] and gepotidacin [14] were tested.

Briefly, HR027 showed high-level resistance to azithromycin, also exhibiting resistance to ceftriaxone, cefixime, ciprofloxacin, tetracycline, and benzylpenicillin, as well as penicillinase production. Nevertheless, the XDR strain was susceptible to spectinomycin and showed wild-type MICs of gentamicin, zoliflodacin [12,13], and gepotidacin [14] (Table).

## Molecular investigation

Sequencing of HR027 was performed with Illumina NextSeq 550 (Illumina, San Diego, CA, United States), as previously described [17], and sequencing reads are publicly available at the European Nucleotide Archive (ENA accession number: PRJEB112818). Quality control and bioinformatic analysis, including characterisation of molecular epidemiological sequence types (STs) and AMR determinants, were performed using the Pathogenwatch platform [18] and a customised CLC Genomics Workbench, as described [17].

Multilocus sequence typing (MLST) of HR027 identified ST9903 and NG sequence typing for antimicrobial resistance (NG-STAR) resulted in a novel ST, namely NG-STAR ST7905 (NG-STAR clonal complex (CC) 7557). The isolate harboured the mosaic *penA*-60.001 allele (encoding the resistance-associated A311V, V316T, and T483S amino acid substitutions in penicillin-binding protein 2) causing resistance to extended-spectrum cephalosporins (ceftriaxone, cefixime), and 23S rRNA A2045G target mutations (corresponding to A2059G in *Escherichia coli*) in all four 23S rRNA gene alleles causing the high-level azithromycin resistance [2,4-7,17,18]. Additional AMR determinants included mutations in *gyrA* (encoding GyrA S91F, D95A) and *parC* (encoding ParC S87N, E91K) genes causing fluoroquinolone resistance, as well as *tetM* and a mutation in the *rpsJ* gene (V57M) causing tetracycline resistance; *bla*_TEM-1_ was also present and responsible for high-level benzylpenicillin resistance [18] (Table). No mutations associated with enhanced MICs of zoliflodacin (GyrB D429 or K450 substitutions [13]) or gepotidacin (GyrA A92 substitution [14]) were found.

We compared the HR027 genome with 76,034 publicly available gonococcal genomes (Figure). While XDR gonococcal strains harbouring *penA*-60.001 and 23S rRNA A2045G mutations and displaying both ceftriaxone and high-level azithromycin resistance have been especially prevalent in Cambodia [8,19], these XDR strains have mainly been of MLST ST16406 and NG-STAR CC5793 [19,20]. The Croatian XDR strain HR027 (MLST ST9903, NG-STAR CC7557) did not cluster with the previously described XDR clone from Cambodia, which belongs to genomic lineage A, a lineage that contains most gonococcal strains with resistance to modern antimicrobials such as ceftriaxone and azithromycin [17,19]. Instead, HR027 clustered with azithromycin-susceptible and mostly ceftriaxone-susceptible strains from Cambodia in genomic lineage B, which has been so far regarded as highly enriched in antimicrobial‑susceptible strains [17,19]. 

**FIGURE:**
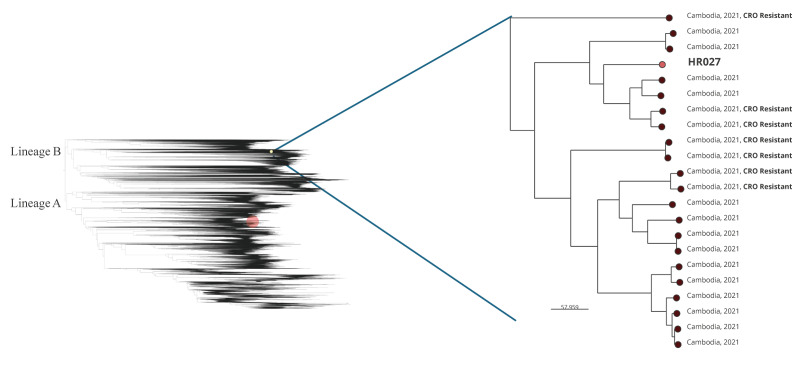
Phylogenetic tree based on publicly available *Neisseria gonorrhoeae* genome sequences and that of the HR027 XDR strain detected in Croatia, with an inset magnifying the 21 most closely related sequences to the HR027 one, Croatia, March 2026 (n = 76,034 genome sequences)

CRO: ceftriaxone; MLST: multilocus sequence typing; NG-STAR: sequence typing for antimicrobial resistance; ST: sequence type; XDR: extensively drug-resistant.

The phylogenetic position of the HR027 strain identified in the current study within the global gonococcal population structure is assessed using publicly available genomes (n = 76,034) analysed in MBioSeq Ridom Typer (Bruker, Billerica, MA, United States). The genomic sequence of the HR027 strain identified in the current study segregates within the genomic lineage B in the global tree shown on the left. The 21 genomes most closely related to the genome of HR027 are shown in the magnified inset on the right. These comprise only genomes from Cambodian isolates. The previously described XDR clone from Cambodia (MLST ST16406, NG-STAR ST5793) [19] in the genomic lineage A, is indicated by the red circle (left tree).

## Discussion

We present the first case of gonorrhoea caused by an XDR NG strain in Croatia. The strain was in vitro resistant to ceftriaxone and high-level resistant to azithromycin, however, the patient was successfully cured with a single 2 g intravenous dose of ceftriaxone. It has also been previously shown, clinically and pharmacodynamically, that ceftriaxone 1 g cures most urogenital infections caused by ceftriaxone-resistant strains with ceftriaxone MICs of 0.25–0.5 mg/L [7,21,22]. Nevertheless, based on observed increasing levels of ceftriaxone resistance and international transmission of XDR strains [2-8,19-21, this study], it appears important to have the novel antimicrobials zoliflodacin [12,13] and gepotidacin [14] licensed and available for use internationally. So far, to the best of our knowledge, all phenotypically and genomically examined XDR strains have shown wild-type MICs of both zoliflodacin and gepotidacin [5,19, this study].

Croatia is participating in the European Gonococcal Antimicrobial Surveillance Programme (Euro-GASP [17,23]), however, very low numbers of cultured gonococcal isolates have been obtained each year (range: 0 to 25 since 2016). Our findings underscore the importance of strengthening the gonococcal culture-based national surveillance and Euro-GASP [17,23] in Croatia, by increasing the number of gonococcal cultures performed and improving the collection of clinical and epidemiological data (including sexual orientation, travel history, and partner notification) across the country. This will allow to improve the detection and monitor the transmission of AMR strains and AMR determinants. It is a major concern that XDR strains, with *penA*-60.001-associated ceftriaxone resistance plus high-level azithromycin resistance, have now evolved in both of the main genomic lineages (A and B) of the gonococcal species phylogeny [17,19]. The prevalence of these XDR strains is high in Cambodia [8,19,20], but these strains are also increasingly detected in European countries [2,4-7], including now Croatia. The location of HR027 in the global gonococcal population suggests that the strain arose from a ceftriaxone-resistant genomic lineage B background already harbouring *penA*-60.001, with subsequent acquisition of 23S rRNA A2045G-mediated high-level azithromycin resistance, not closely related genomically with the previously described XDR clone in Cambodia [19]. 

Increased awareness is of utmost importance among clinicians, including those working at the primary healthcare level, regarding presence of XDR gonococcal strains in Croatia and internationally, and the need to adhere to national and international recommendations concerning prevention, diagnostics and treatment (including follow-up and test-of-cure) [1]. Enhanced awareness should also target populations at higher risk of gonorrhoea, including men who have sex with men engaging in high-risk sexual behaviours, in particular in light of the expansion of HIV pre-exposure prophylaxis in Croatia and internationally, as well as heterosexual individuals prone to high-risk sexual behaviours [24]. Actions to increase the awareness and adherence to up-to-date evidence-based guidelines for management (sampling, diagnosis and treatment) of gonorrhoea [1] have been initiated in Croatia.

The limitations of the present study included that the female partner could not be traced and that extragenital samples were not initially collected and tested for the male patient, which is important to perform in XDR gonorrhoea cases.

## Conclusions

Our findings of an XDR gonococcal strain in Croatia highlight the expanding international concern posed by XDR strains combining ceftriaxone resistance (caused by mosaic *penA*-60.001 allele) with high-level azithromycin resistance (caused by 23S rRNA A2045G mutation in all four gene alleles). Intensified and improved gonorrhoea management and surveillance (including prevention, timely diagnosis, treatment, and reporting) are imperative in Croatia and internationally, and integrated AMR monitoring and genomic surveillance represent key tools for early detection of AMR, guidance of therapeutic strategies, and improved public health.

## Data Availability

The first author (TJ) and the last author (MU) had full access to all the data in the study and datasets can be made available from the corresponding author after publication on reasonable request. All sequencing reads are publicly available at the European Nucleotide Archive (ENA accession number: PRJEB112818).
